# Digital and Smart Technologies for Early Pressure Injury Detection and Prevention in Stroke and Mobility‐Impaired Patients: A Scoping Review

**DOI:** 10.1155/nrp/6992856

**Published:** 2026-08-12

**Authors:** Muhammad Abu, Aulia Insani Latif, Jenita Laurensia Saranga, Zakariyati, Evi Kusmayanti, Nur Halimah, Deliaty Bagenda Ali, Ruqaiyah

**Affiliations:** ^1^ Department of Medical Surgical Nursing, Pelamonia Institute of Health Sciences, Jl. Garuda No. 3AD Kunjung Mae, Mariso Makassar, 90113, Indonesia; ^2^ Department of Medical Surgical Nursing, Faculty of Nursing, Hasanuddin University, Jl. Perintis Kemerdekaan Km. 10 Tamalanrea, Makassar 90245, Indonesia, unhas.ac.id; ^3^ Department of Pediatric Nursing, Pelamonia Institute of Health Sciences, Jl. Garuda No. 3AD Kunjung Mae Mariso, Makassar 90113, Indonesia; ^4^ Department of Maternity Nursing, Pelamonia Institute of Health Sciences, Jl. Garuda No. 3 AD Kunjung Mae Mariso, Makassar 90113, Indonesia; ^5^ Department of Midwifery, Pelamonia Institute of Health Sciences, Jl. Garuda No. 3AD Kunjung Mae Mariso, Makassar 90113, Indonesia

**Keywords:** digital health, immobilization, pressure injury prevention, smart technology, stroke

## Abstract

**Background:**

Pressure injuries remain a major complication among stroke and other mobility‐impaired populations. Digital and smart technologies are increasingly used to support early tissue‐risk detection, monitoring, and prevention‐related care processes. However, evidence remains dispersed across technology types, clinical functions, and care settings.

**Methods:**

This scoping review followed the Joanna Briggs Institute methodology and PRISMA‐ScR guidance. PubMed/MEDLINE, ScienceDirect, CINAHL Complete via EBSCOhost, IEEE Xplore, and Wiley Online Library were searched for English‐language studies published from 1 January 2018 to 30 July 2025. Eligible studies involved digital or smart technologies used for early pressure injury detection, physiological risk monitoring, repositioning support, pressure redistribution, or prevention‐related decision support in stroke or other mobility‐impaired populations. Two reviewers screened records using Rayyan and charted study characteristics, technology functions, settings, and outcomes. Critical appraisal was not undertaken because the review aimed to map evidence rather than estimate effects.

**Results:**

Of 1063 identified records, 13 studies were included. Eight technology categories were mapped: subepidermal moisture scanners, pressure ulcer monitoring platform devices, comprehensive mobile assessment of pressure, artificial intelligence‐enabled smartphone applications, artificial intelligence‐powered smart decompression mattresses, Internet of Things‐based smart bed systems, infrared thermography, and digital skin moisture monitoring. Subepidermal moisture scanners were the only technology represented by multiple studies (6/13); all other categories were represented by single studies. Reported outcomes mainly involved diagnostic support, repositioning adherence, feasibility, and workflow‐related measures. Direct evidence on pressure injury incidence was limited and heterogeneous. Most studies were hospital‐based, with very limited community or home‐care evidence.

**Conclusion:**

Digital and smart technologies may support earlier recognition of pressure‐related tissue changes and selected prevention‐related care processes, particularly in hospitals. However, evidence remains heterogeneous and insufficient to support strong effectiveness claims across technology types.

**Implications:**

Future research should prioritize clear prevention outcomes, comparative and implementation studies, and community‐based testing before considering meta‐analysis.

## 1. Introduction

Stroke remains one of the most serious global health problems, occurring when the blood supply to the brain is disrupted and resulting in neuronal cell damage [1]. According to the World Health Organization, stroke is the second leading cause of death worldwide and a major contributor to long‐term disability among adults [2]. Beyond acute neurological deficits, stroke frequently leads to persistent motor and sensory impairments that substantially reduce functional independence [3]. As a consequence, many stroke survivors experience prolonged immobility due to paralysis or diminished self‐mobility [4]. This condition is often compounded by sensory loss, which limits the ability to perceive pressure or discomfort and markedly increases the risk of pressure injury development [5].

Pressure injuries are localized damage to the skin and underlying tissue that typically occur over bony prominences due to sustained pressure, friction, or shear forces [6]. These injuries are associated with pain, delayed recovery, and a substantial burden on healthcare systems [7]. Patients who develop pressure injuries often require complex wound care, experience prolonged hospitalization, and face serious complications, including infection and sepsis [8]. Pressure injuries are particularly prevalent among stroke patients and individuals with severe immobility who remain bedridden for extended periods and are unable to reposition independently [9]. Prolonged pressure disrupts local blood flow, depriving tissues of oxygen and nutrients and leading to erythema, tissue breakdown, and ulcer formation that is highly susceptible to infection [10]. In the long term, pressure injuries significantly reduce quality of life and are associated with increased morbidity, mortality, and healthcare costs [11].

Given the high vulnerability of stroke and immobilized patients, timely, structured, and prevention‐oriented strategies are essential. Pressure injury prevention is widely recognized as a key indicator of nursing care quality [12]. Multiple contributing factors have been identified, including immobility, sensory impairment, malnutrition, skin moisture, friction, and shear forces [13]. The economic impact of pressure injuries is considerable, with estimated treatment costs ranging from USD 20,900 to USD 151,700 per patient per case [14]. Beyond the physical burden, pressure injuries also adversely affect psychosocial well‐being, contributing to chronic pain, emotional distress, social isolation, and depression [15].

In this review, pressure injury prevention is defined as a continuum of activities used to identify elevated risk or early tissue compromise and to trigger actions intended to prevent incident injury or progression of pressure‐related tissue damage [16]. This continuum includes structured risk assessment, skin and tissue assessment, physiological monitoring, repositioning support, pressure redistribution, and prevention‐related clinical decision support. This framing is important because digital and smart technologies do not all operate at the same point in the prevention pathway. Some technologies are designed to detect subclinical tissue changes before visible skin damage occurs, some monitor preventive care processes such as repositioning, and others support pressure redistribution or nursing decision‐making [17–22].

Digital and smart technologies have increasingly been introduced to support pressure injury prevention and early risk recognition. In the studies included in this review, these technologies included subepidermal moisture (SEM) scanners, repositioning monitoring platforms, mobile pressure assessment systems, artificial intelligence‐enabled smartphone applications, smart decompression mattresses, Internet of Things‐based smart beds, infrared thermography, and digital skin moisture monitoring devices [7, 17–28]. However, the existing evidence remains dispersed across technology types, patient groups, care settings, and outcome measures. This dispersion makes it difficult to determine how these technologies function within the prevention pathway and where the strongest and weakest areas of evidence are located.

Recent reviews have begun to examine eHealth, digital, and self‐managed technologies for pressure injury prevention in broader populations, including individuals with spinal cord injury and those receiving long‐term care. Nevertheless, the position of stroke‐focused and mobility‐impaired populations within this broader evidence base remains insufficiently mapped. In addition, many technologies are reported in single studies, and their functions vary from early tissue‐risk detection to monitoring, pressure redistribution, and decision support. Accordingly, this scoping review aims to map digital and smart technologies used within the pressure injury prevention pathway for stroke patients and patients with mobility impairment, with emphasis on technology function, implementation context, and evidence gaps.

## 2. Objectives

The objective of this scoping review was to map digital and smart technologies used within the pressure injury prevention pathway for adults with stroke or mobility impairment and to describe their functions, care settings, and remaining evidence gaps.

In this review, the pressure injury prevention pathway was understood as a continuum that includes early tissue‐risk detection, physiological risk monitoring, repositioning support, pressure redistribution, and prevention‐related clinical decision support.

The PCC framework was defined as follows:

a. Population (P): adults with stroke or mobility impairment who were at risk of pressure injury.
b. Concept (C): digital or smart technologies used within the pressure injury prevention pathway, including technologies for early tissue‐risk detection, physiological risk monitoring, repositioning support, pressure redistribution, or prevention‐related decision support.
c. Context (C): pressure injury prevention in hospital, rehabilitation, long‐term care, community, or home‐care settings.

This review addressed four questions:

a. Which digital and smart technologies have been reported for pressure injury prevention or early prevention‐related assessment in adults with stroke or mobility impairment?
b. What primary prevention‐related function does each technology serve within the pressure injury prevention pathway?
c. In which care settings have these technologies been studied?
d. What evidence gaps remain across populations, technology functions, care settings, and reported outcomes?

## 3. Methods

### 3.1. Design

This scoping review was conducted in accordance with the Joanna Briggs Institute methodology for scoping reviews and reported using the Preferred Reporting Items for Systematic Reviews and Meta‐Analyses extension for Scoping Reviews (PRISMA‐ScR) [29, 30]. The completed PRISMA‐ScR checklist is provided in Supporting File S2. A scoping review approach was selected because the purpose of this study was to map the range, functions, contexts, and gaps in the available evidence rather than to estimate intervention effectiveness.

### 3.2. Protocol and Registration

No publicly accessible protocol or registration record was available for this review. The methods were developed a priori by the review team and are reported here in full. The review was not registered in PROSPERO because it was designed as a scoping review.

### 3.3. Search Strategy and Information Sources

The databases searched were PubMed/MEDLINE, ScienceDirect, CINAHL Complete via EBSCOhost, IEEE Xplore, and Wiley Online Library. Searches were limited to English‐language records published from January 1, 2018, to July 30, 2025. January 2018 was selected as the start date to capture contemporary digital, mobile, sensor‐based, and artificial intelligence‐enabled technologies with likely relevance to current clinical implementation. Reference lists of included articles were checked manually to identify potentially relevant additional records. However, no additional studies meeting the eligibility criteria were identified through this process; therefore, no additional records from citation searching were added to the PRISMA‐ScR flow diagram.

The search strategy combined terms related to digital or smart technology, pressure injury prevention, and mobility‐impaired populations. Search terms included “digital technology,” “smart technology,” “artificial intelligence,” “mobile health,” “wearable devices,” “monitoring system,” “pressure injury,” “pressure ulcer,” “bedsore,” “stroke patients,” “immobilized patients,” “mobility impairment,” “prevention,” and “risk assessment.” The full PubMed/MEDLINE search strategy and database‐specific adaptations are provided in Supporting Table S1.

### 3.4. Eligibility Criteria

Eligible studies involved adults with stroke or impaired mobility who were at risk of pressure injury and reported original empirical data on digital or smart technologies used for early tissue‐risk detection, physiological risk monitoring, repositioning support, pressure redistribution, or prevention‐related decision support.

Digital or smart technologies were defined as electronic, sensor‐based, mobile, artificial intelligence‐enabled, Internet of Things‐based, imaging‐based or device‐assisted technologies designed to support assessment, monitoring, decision‐making, repositioning, or pressure redistribution within the pressure injury prevention pathway.

Quantitative, qualitative, mixed‐methods, quasiexperimental, clinical evaluation, prospective, retrospective, and observational studies were eligible. Reviews, editorials, commentaries, protocols, conference abstracts without sufficient data, studies without full‐text availability, and studies focused solely on nondigital care or wound treatment without a prevention‐related monitoring or assessment component were excluded.

### 3.5. Study Selection

Study selection was managed using Rayyan. Two reviewers independently screened titles and abstracts against the eligibility criteria. Potentially relevant articles were retrieved in full text and assessed for final eligibility. Disagreements were resolved through discussion or consultation with a third reviewer when necessary. Inter‐rater agreement for title and abstract screening was substantial (*κ* = 0.82).

### 3.6. Data Charting

Data charting was conducted using a modified Joanna Briggs Institute charting form. The charting form captured author and year, country, study aim, study design, population and sample size, care setting, type of technology, primary prevention‐related function, reported outcomes, and implementation notes. The charting form was discussed by the review team before use to ensure consistency in data extraction.

### 3.7. Critical Appraisal

Formal critical appraisal of included studies was not undertaken. This decision was consistent with the purpose of the review, which was to map the breadth and characteristics of the available evidence rather than to determine the effectiveness of interventions or grade the certainty of evidence. Therefore, reported findings should be interpreted as descriptive mapping rather than comparative effectiveness estimates.

### 3.8. Data Synthesis

Data were synthesized descriptively and thematically. Descriptive synthesis was used to summarize publication year, country, care setting, study design, sample size, population, and technology category. To strengthen synthesis beyond study‐by‐study description, technologies were grouped according to their dominant prevention‐related function. This function‐based synthesis generated four themes: objective early tissue‐risk detection, artificial intelligence‐enabled rapid assessment and decision support, smart platforms for repositioning and pressure redistribution, and noninvasive physiological monitoring adjuncts. The synthesis focused on mapping technology functions, implementation contexts, reported outcome domains, and evidence gaps across the included studies.

## 4. Results

### 4.1. Study Selection

**FIGURE 1:**
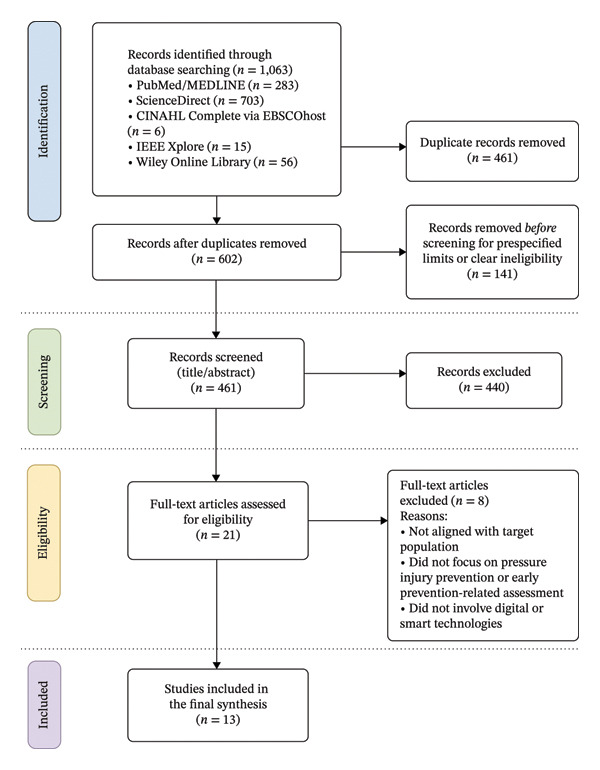
PRISMA‐ScR flow diagram.

A total of 1063 records were identified from the database searches. After removal of duplicates, 602 unique records remained. A further 141 records were removed before screening because they did not meet prespecified limits or were clearly ineligible at import. Thus, 461 records proceeded to title and abstract screening. Of these, 440 records were excluded because they did not meet the eligibility criteria. Twenty‐one full‐text articles were assessed for eligibility, and eight were excluded because they were not aligned with the target population, did not focus on pressure injury prevention or early prevention‐related assessment, or did not involve digital or smart technologies. Thirteen studies were included in the final synthesis. The study selection process is presented in Figure 1.

### 4.2. Characteristics of Included Studies

**FIGURE 2:**
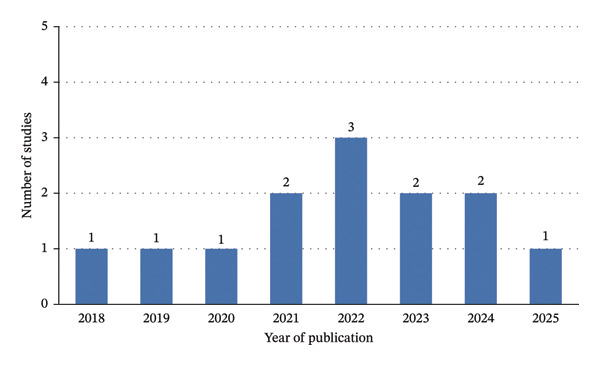
Trend of publications on digital and smart technologies for pressure injury prevention in stroke and mobility‐impaired patients (2018–2025).

The 13 included studies were published between 2018 and 2025. The characteristics of the included studies, including country, study design, population, care setting, technology type, dominant prevention‐related function, key findings, and prevention‐related implications, are presented in Table 1. The publication trend across the review period is shown in Figure 2, with the highest number of included studies published in 2022. The evidence base was small and unevenly distributed across technology types, populations, and care settings. Most studies were conducted in hospital or acute‐care settings, while evidence from community and home‐care contexts was limited. Stroke‐specific evidence was also limited, with several studies involving broader high‐risk or mobility‐impaired populations rather than stroke patients alone.

**TABLE 1:** Characteristics of the included studies and data charting summary.

No.	Author (Year)	Country	Study aim	Study design	Population/sample/setting	Technology type	Dominant prevention‐related function	Key findings	Prevention‐related implications
1	Raizman et al. [21]	Canada	To evaluate the clinical utility of the subepidermal moisture (SEM) scanner.	Quasiexperimental, two‐phase study	284 participants/hospital setting	SEM scanner	Objective early tissue‐risk detection	SEM scanner use was associated with a marked reduction in hospital‐acquired pressure ulcers during the study period.	SEM measurement may support targeted prevention strategies in hospital settings, although implementation should consider workflow integration and staff training.
2	Smith [27]	United Kingdom	To evaluate the use of SEM scanning to detect nonvisible pressure damage and support targeted pressure ulcer prevention interventions.	Clinical practice evaluation	35 patients/hospital setting	SEM scanner	Objective early tissue‐risk detection	SEM scanning supported earlier identification of nonvisible pressure damage and targeted repositioning.	SEM measurement may assist early risk recognition and targeted prevention planning in hospital settings.
3	Olney et al. [20]	United States	To report iterative redesign and assess the feasibility and utility of the comprehensive mobile assessment of pressure system for pressure injury prevention in veterans with spinal cord injury.	Mixed‐methods study	18 veterans with spinal cord injury using manual or electric wheelchairs/community setting	CMAP mobile application	Repositioning support and pressure mapping	Real‐time pressure mapping and repositioning reminders were perceived as useful and easy to use by veterans with spinal cord injury.	CMAP may support pressure awareness, repositioning adherence, and self‐management among wheelchair users, but further studies are needed to evaluate its effect on pressure injury outcomes.
4	Minteer et al. [18]	United States	To validate two prototypes of the pressure ulcer monitoring platform for monitoring repositioning among inpatients.	Prospective clinical experimental study	10 hospitalized patients with limited mobility/hospital setting	PUMP devices	Repositioning monitoring	The PUMP devices recorded patient repositioning activity with approximately 85% reliability in hospitalized patients with limited mobility.	PUMP may support objective monitoring of repositioning activity and inform pressure injury prevention workflows, but further validation in larger clinical samples is needed.
5	Okonkwo et al. [19]	United States and United Kingdom	To evaluate SEM as a biomarker for early pressure injury detection.	Prospective study	189 inpatients/hospital setting	SEM biocapacitance device	Objective early tissue‐risk detection	SEM biocapacitance measurement showed potential for earlier detection of pressure injury risk compared with traditional clinical assessment.	SEM may complement visual skin assessment in high‐risk inpatients.
6	Nightingale and Musa [26]	United Kingdom	To evaluate the impact of adding SEM scanning to the standard care pathway on hospital‐acquired pressure ulcer incidence.	Clinical evaluation study	697 patients/hospital setting	SEM scanner	Objective early tissue‐risk detection	Adding SEM scanning to the standard care pathway was associated with lower Category 2–4 pressure ulcer incidence in the evaluated hospital setting.	SEM scanning may support early detection within hospital‐based pressure injury prevention pathways.
7	Lau et al. [17]	China	To evaluate the accuracy of an artificial intelligence‐enabled smartphone application for real‐time pressure injury assessment.	Experimental image‐based study	144 wound images/hospital‐related image dataset	AI‐enabled smartphone application	Rapid assessment and decision support	The AI‐enabled smartphone application achieved an overall prediction accuracy of 63.2% for pressure injury image assessment.	AI‐enabled image assessment may support rapid classification and decision support, but further validation is required before broad clinical use.
8	Abu et al. [23]	Indonesia	To assess repositioning for pressure ulcer risk prevention using digital skin moisture monitoring.	Quasiexperimental, nonequivalent control group design	60 patients, 30 intervention and 30 control/hospital setting	Digital skin moisture monitoring	Physiological skin monitoring and repositioning support	Repositioning guided by digital skin moisture assessment was associated with improved prevention‐related outcomes.	Digital skin moisture monitoring may support nursing decision‐making for repositioning, but further evaluation is needed.
9	Byrne et al. [24]	Ireland	To investigate SEM monitoring and targeted pressure ulcer prevention compared with visual skin assessment and usual care.	Quasiexperimental observational study	238 at‐risk patients/Acute hospital setting	SEM Scanner	Objective early tissue‐risk detection	SEM monitoring combined with targeted prevention was associated with improved early identification compared with visual assessment alone.	SEM measurement may support targeted prevention planning in acute hospital settings.
10	Yang et al. [22]	China	To examine an AI‐powered smart decompression mattress for preventing moderate‐ and high‐risk pressure injuries in postoperative geriatric patients.	Retrospective cohort analysis	400 postoperative geriatric patients/Hospital setting	AI‐powered smart decompression mattress	Pressure redistribution	Use of the AI‐powered smart decompression mattress was associated with reduced pressure injury severity, fewer complications, and improved sleep quality in the study population.	Smart decompression mattresses may support pressure redistribution in postoperative older adults, but comparative evidence remains limited.
11	Wilson et al. [7]	Ireland	To explore correlations between SEM, epidermal hydration, temperature, pain, and ultrasound as early indicators of pressure ulcer development.	Prospective cohort observational study	60 participants/hospital setting	Provizio SEM Scanner™ and ultrasound	Objective early tissue‐risk detection	SEM measurement showed significant correlations with ultrasound and other early indicators of pressure ulcer development.	SEM may assist early tissue‐risk assessment, but further studies are needed to clarify its role as a standalone tool.
12	Taryudi et al. [28]	Indonesia	To evaluate an IoT‐enabled smart bed system for pressure ulcer prevention among patients with stroke.	Quasiexperimental study	30 stroke patients, 15 intervention, and 15 control/hospital setting	IoT‐enabled smart bed system	Pressure redistribution and monitoring	The IoT‐enabled smart bed group showed improvement in selected pressure injury risk indicators among stroke patients.	IoT‐based smart beds may support pressure redistribution and monitoring in stroke care, but larger studies are needed.
13	Lin et al. [25]	China	To examine the accuracy of infrared thermography in predicting pressure injury healing outcomes.	Prospective study	156 adults with pressure injuries/hospital setting	Infrared thermography application	Noninvasive physiological monitoring adjunct	Relative wound temperature was associated with pressure injury healing outcomes; day‐one temperature was reported as a significant predictor.	Infrared thermography may serve as an adjunct physiological assessment tool, but its role in primary prevention requires further clarification.

*Abbreviations:* AI, artificial intelligence; CMAP, comprehensive mobile assessment of pressure; IoT, Internet of Things; PUMP, pressure ulcer monitoring platform; SEM, subepidermal moisture.

The included studies used heterogeneous designs, including quasiexperimental studies, prospective observational studies, clinical practice evaluations, mixed‐methods studies, retrospective cohort analysis, and experimental image‐based assessment. Sample sizes varied substantially across studies, ranging from small feasibility studies to larger hospital‐based evaluations. This variation limited direct comparison across technologies and reinforced the need to interpret the findings as evidence mapping rather than effectiveness estimation.

**FIGURE 3:**
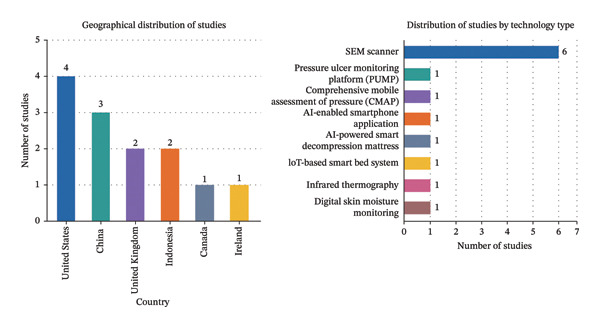
Distribution of included studies by geography and technology type (2018–2015).

Across the included studies, eight categories of digital or smart technologies were identified: SEM scanners, pressure ulcer monitoring platform devices, comprehensive mobile assessment of pressure, artificial intelligence‐enabled smartphone applications, artificial intelligence‐powered smart decompression mattresses, Internet of Things‐based smart bed systems, infrared thermography, and digital skin moisture monitoring. SEM scanners were the most frequently represented technology, appearing in six studies. All other technology categories were represented by single studies. The geographical distribution of the included studies and the distribution of studies by technology type are summarized in Figure 3.

### 4.3. Technology Categories and Prevention‐Related Functions

The technologies identified in this review did not operate at the same point in the pressure injury prevention pathway. Therefore, they were mapped according to their dominant prevention‐related function rather than being interpreted as equivalent interventions. Across the included studies, technologies were positioned within four broad functional areas: early tissue‐risk detection, artificial intelligence‐enabled rapid assessment and decision support, repositioning or pressure redistribution support, and noninvasive physiological monitoring. This function‐based organization was used to guide the thematic synthesis presented below.

### 4.4. Function‐Based Thematic Synthesis

**FIGURE 4:**
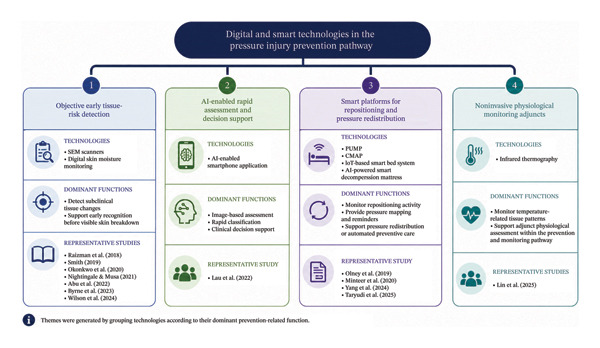
Function‐based thematic synthesis of digital and smart technologies.

The function‐based synthesis generated four themes: objective early tissue‐risk detection, artificial intelligence‐enabled rapid assessment and decision support, smart platforms for repositioning and pressure redistribution, and noninvasive physiological monitoring adjuncts. Figure 4 presents the function‐based thematic synthesis, showing how the included technologies were grouped according to their dominant prevention‐related function within the pressure injury prevention pathway.

### 4.5. Theme 1: Objective Early Tissue‐Risk Detection

The first theme involved technologies designed to support objective detection of early tissue‐risk indicators before or alongside visible skin changes. This theme was represented mainly by SEM scanners and digital skin moisture monitoring [7, 19, 21, 23, 24, 26, 27]. Subepidermal moisture scanners were the most frequently studied technology in this review, appearing in six of the thirteen included studies. Across these studies, SEM measurement was reported as a tool that may support earlier recognition of subclinical tissue changes compared with visual skin inspection alone. However, the strength of evidence varied across study designs, and the findings should not be interpreted as pooled effectiveness evidence.

Digital skin moisture monitoring was represented by one study and was used to guide prevention‐related nursing care, particularly repositioning. Together, these findings suggest that moisture‐related technologies occupy an important position in the early detection component of the prevention pathway. Nevertheless, further comparative and implementation‐focused studies are needed to clarify how these tools should be integrated into routine nursing assessment, especially outside hospital settings.

### 4.6. Theme 2: Artificial Intelligence‐Enabled Rapid Assessment and Decision Support

The second theme included artificial intelligence‐enabled technologies used to support rapid assessment, classification, or decision support. One study evaluated an artificial intelligence‐enabled smartphone application for real‐time pressure injury assessment using wound images [17]. This technology was primarily related to classification and decision support rather than direct prevention of incident pressure injury.

The available evidence suggests that artificial intelligence‐enabled assessment tools may have potential value for supporting rapid clinical judgment, documentation, or remote assessment. However, because this technology was represented by a single study and focused mainly on image‐based classification, its role within the prevention pathway remains preliminary. Further research is needed to evaluate accuracy, clinical workflow integration, usability, and whether artificial intelligence‐supported assessment can meaningfully improve prevention‐related outcomes.

### 4.7. Theme 3: Smart Platforms for Repositioning and Pressure Redistribution

The third theme involved technologies that supported repositioning monitoring, pressure redistribution, or automated preventive care processes. This theme included the pressure ulcer monitoring platform, comprehensive mobile assessment of pressure, Internet of Things‐based smart bed systems, and artificial intelligence‐powered smart decompression mattresses [18, 20, 22, 28].

These technologies were generally designed to address mechanical risk factors for pressure injury, particularly sustained pressure and inadequate repositioning. The pressure ulcer monitoring platform focused on monitoring repositioning activity among hospitalized patients. The comprehensive mobile assessment of pressure provided real‐time pressure mapping and repositioning reminders among community‐dwelling wheelchair users. Internet of Things‐based smart bed systems and artificial intelligence‐powered smart decompression mattresses were used to support pressure redistribution or automated repositioning‐related care.

Although these technologies are conceptually aligned with pressure injury prevention, the evidence remains limited because each technology type was represented by a small number of studies or a single study. Reported outcomes were heterogeneous and included repositioning adherence, sensory perception, pressure injury risk indicators, sleep quality, complications, and feasibility. Therefore, these findings should be interpreted as preliminary evidence of possible prevention‐related utility rather than definitive evidence of effectiveness.

### 4.8. Theme 4: Noninvasive Physiological Monitoring Adjuncts

The fourth theme involved noninvasive physiological monitoring technologies used to support assessment of tissue or wound status. Infrared thermography was represented by one study and was used to assess temperature‐related patterns associated with pressure injury healing [25]. Although this technology may provide useful physiological information, its role in primary prevention remains less direct than technologies designed to detect early tissue risk or support repositioning and pressure redistribution.

Infrared thermography may be more appropriately understood as an adjunctive assessment tool within the broader prevention and monitoring pathway, particularly for identifying changes in tissue condition or healing potential. Further research is needed to clarify whether thermographic assessment can support earlier prevention‐related decisions before pressure injuries progress.

### 4.9. Evidence Gaps

Several evidence gaps were identified across the included studies. First, the evidence was concentrated in hospital settings, with limited research in rehabilitation, long‐term care, community, and home‐care contexts. Second, most technology categories were represented by single studies, except SEM scanners. Third, outcome measures were heterogeneous, including pressure injury incidence, repositioning activity, assessment accuracy, feasibility, workflow outcomes, and healing‐related measures. Fourth, stroke‐specific evidence remained limited because several studies involved broader high‐risk or mobility‐impaired populations. Finally, implementation outcomes, including cost, training needs, usability, staff workload, data security, patient acceptability, and integration into routine nursing care, were inconsistently reported.

## 5. Discussion

This scoping review mapped digital and smart technologies used within the pressure injury prevention pathway for stroke patients and other mobility‐impaired populations. The findings show that the available evidence is still limited, heterogeneous, and unevenly distributed across technology types, care settings, and patient populations. SEM scanners were the most frequently represented technology, whereas most other technologies, including artificial intelligence‐enabled smartphone applications, smart decompression mattresses, Internet of Things‐based smart beds, repositioning monitoring platforms, infrared thermography, and digital skin moisture monitoring, were represented by single studies [7, 17–28]. This pattern suggests that the field is developing, but the current evidence is not yet sufficient to support strong comparative or effectiveness conclusions across technology types.

A central finding of this review is that digital and smart technologies do not contribute to pressure injury prevention in the same way. Rather, they appear to operate at different points within the prevention pathway. Some technologies support early tissue‐risk detection, some monitor preventive care processes, some assist pressure redistribution or repositioning, and others support image‐based assessment or physiological monitoring [17, 18, 20, 22, 25, 28]. This distinction is important because prevention should not be interpreted only as reduction in pressure injury incidence. In high‐risk populations such as stroke patients and mobility‐impaired adults, prevention may also involve identifying early tissue compromise, supporting timely repositioning, monitoring physiological risk indicators, and informing clinical decision‐making before visible skin breakdown occurs.

The strongest concentration of evidence was found for SEM scanners. Across the included studies, SEM measurement was reported as a technology that may support earlier recognition of subclinical tissue changes compared with visual inspection alone [7, 19, 21, 24, 26, 27]. This finding is consistent with previous evidence suggesting that SEM may be a useful early indicator of pressure‐related tissue change [31, 32]. However, even for SEM scanners, the evidence should be interpreted carefully. The included studies varied in design, population, setting, comparator, and outcome measurement. Therefore, the findings indicate potential clinical utility, particularly for early tissue‐risk recognition, but they do not provide pooled evidence of effectiveness.

Other technologies were less frequently studied and should be understood as emerging or preliminary areas of evidence. Repositioning and pressure‐related technologies, such as the pressure ulcer monitoring platform, comprehensive mobile assessment of pressure, Internet of Things‐based smart beds, and artificial intelligence‐powered smart decompression mattresses, were conceptually aligned with prevention because they addressed mechanical risk factors such as sustained pressure, inadequate repositioning, or pressure redistribution [18, 20, 22, 28]. Nevertheless, these technologies were represented by limited numbers of studies and used different outcome measures, including repositioning activity, feasibility, sensory perception, sleep quality, complications, or pressure injury risk indicators. As a result, their role in reducing pressure injury incidence remains uncertain and requires further comparative evaluation.

Artificial intelligence‐enabled assessment tools also appeared in the evidence base, but their current contribution was mainly related to rapid assessment, image‐based classification, or decision support rather than direct prevention of incident pressure injury [17]. This distinction is important because classification accuracy or image‐recognition performance should not be equated with prevention effectiveness. AI‐enabled tools may support clinical documentation, triage, remote assessment, and decision‐making, but future studies need to determine whether these tools improve prevention‐related outcomes, reduce delays in care, or support nurses in identifying patients who require targeted preventive interventions [33, 34].

Infrared thermography and digital skin moisture monitoring represent additional forms of noninvasive physiological or skin‐related assessment. These technologies may provide objective information on tissue condition, skin moisture, or temperature‐related changes [23]. Their potential value lies in supporting more objective assessment of early risk or wound‐related status. However, the evidence remains limited, and their role within primary pressure injury prevention requires clearer evaluation. Future studies should distinguish whether these tools are being used for early risk recognition, monitoring of existing injury, prediction of healing, or prevention of progression [32, 35].

Building on the mapped evidence gaps, the findings highlight important limitations in population focus and care settings. Although the title and focus of this review include stroke patients, stroke‐specific studies were limited. Several included studies involved broader high‐risk groups, such as hospitalized adults, surgical patients, older adults, individuals with spinal cord injury, or other mobility‐impaired populations [18, 20, 22]. This reflects the broader nature of pressure injury risk but also indicates that evidence specific to stroke care remains underdeveloped. In addition, most studies were conducted in hospital settings. Evidence from rehabilitation units, long‐term care, community care, and home‐care settings was scarce, despite the fact that many stroke survivors and mobility‐impaired patients remain at risk after discharge [9].

Implementation issues were also inconsistently addressed across the included studies. Digital and smart technologies require more than technical performance; they also require feasibility within real clinical workflows. Staff training, usability, cost, maintenance, interoperability, patient acceptability, data privacy, and integration into nursing documentation are critical factors for implementation. These issues are particularly important for resource‐limited settings, where access to advanced devices may be constrained by cost, infrastructure, workforce capacity, and institutional readiness. Literature on digital health and Internet of Things‐based healthcare has similarly emphasized that privacy, security, usability, and implementation context are central to responsible adoption of digital technologies in clinical practice [36–38].

Overall, this review suggests that digital and smart technologies may contribute to pressure injury prevention by supporting earlier recognition of risk, improving objectivity of assessment, monitoring prevention‐related care processes, and assisting decision‐making. However, the evidence base remains fragmented. The current findings should therefore be interpreted as a map of available evidence and knowledge gaps rather than proof of effectiveness. Stronger evidence will require studies with clearer definitions of prevention outcomes, more consistent outcome measures, comparative designs, longer follow‐up, and greater attention to implementation in diverse care settings.

## 6. Clinical and Research Implications

The clinical implications of this review should be interpreted cautiously. Among the technologies identified, SEM scanners have the most developed evidence base and may be the most clinically relevant technology for supporting early tissue‐risk recognition in hospital settings. However, even this evidence does not eliminate the need for careful clinical judgment, staff training, and integration with existing pressure injury prevention protocols.

For other technologies, including AI‐enabled smartphone applications, smart beds, pressure monitoring platforms, infrared thermography, and digital skin moisture monitoring, the evidence is better understood as preliminary and hypothesis‐generating. These technologies may offer useful support for assessment, monitoring, repositioning, or decision‐making, but current evidence is insufficient to recommend broad clinical implementation across settings.

The main implication is therefore directed toward research and implementation planning. Future studies should clearly define the prevention‐related function of each technology, distinguish early detection from prevention of occurrence and prevention of progression, and use outcome measures that are clinically meaningful and comparable across studies. Studies should also evaluate feasibility, usability, cost, training requirements, data security, and workflow integration, particularly in rehabilitation, long‐term care, community, and home‐care settings.

## 7. Limitations

This review has several limitations. First, the search was limited to five electronic databases and did not include gray literature, which may have resulted in the omission of relevant reports, technical evaluations, or implementation documents. Second, only English‐language studies published from 2018 to 2025 were included, which may have excluded relevant studies published in other languages or before the selected time frame. Third, formal critical appraisal was not undertaken because this review aimed to map the breadth and characteristics of the evidence rather than assess intervention effectiveness. Therefore, the findings should not be interpreted as evidence of comparative effectiveness or certainty of effect. Fourth, the included studies were heterogeneous in terms of design, population, care setting, technology type, and outcome measures, which limited a direct comparison across technologies. Finally, several technologies were represented by only one study, and stroke‐specific evidence was limited.

## 8. Conclusion

This scoping review mapped digital and smart technologies used within the pressure injury prevention pathway for stroke patients and other mobility‐impaired populations. The included studies showed that these technologies may support different prevention‐related functions, including early tissue‐risk detection, physiological monitoring, repositioning support, pressure redistribution, image‐based assessment, and clinical decision support. SEM scanners were the most frequently studied technology, while most other technology categories were represented by single studies.

The current evidence suggests potential roles for digital and smart technologies in supporting pressure injury prevention‐related care processes, particularly early recognition of tissue risk in hospital settings. However, the evidence remains limited and heterogeneous, and it is not sufficient to support strong claims of effectiveness across technology types. Future research should prioritize clearly defined prevention outcomes, comparative and implementation‐focused designs, consistent outcome measures, and evaluation in rehabilitation, long‐term care, community, and home‐care settings.

## Author Contributions

Muhammad Abu contributed to the conceptualization and design of the study, literature search, data extraction, thematic synthesis, and drafted the initial manuscript. Evi Kusmayanti and Jenita Laurensia Saranga contributed to data interpretation, methodological input, and critical revision of the manuscript for important intellectual content. Deliaty Bagenda Ali and Ruqaiyah contributed to methodological refinement, interpretation of findings, and substantive review of the manuscript. Zakariyati and Nur Halimah contributed to data screening support, interpretation of results, and critical feedback on the manuscript draft. Aulia Insani Latif as the corresponding author contributed to study supervision, methodological validation, coordination of the revision process, and critical revision of the manuscript.

## Funding

This study was funded by the Ministry of Higher Education, Science, and Technology of the Republic of Indonesia (Grant No. 0419/C3/DT.05.00/2025).

## Disclosure

The funder had no role in the design, search process, data charting, synthesis, interpretation, or writing of this scoping review. All authors reviewed and approved the final version of the manuscript and agree to be accountable for all aspects of the work.

## Ethics Statement

This study is a scoping review using previously published data.

## Conflicts of Interest

The authors declare no conflicts of interest.

## Supporting Information

Additional supporting information can be found online in the Supporting Information section.

## Supporting information


**Supporting Information 1** Supporting Table S1 provides the full search strategy and database‐specific adaptations used in this scoping review, including the PCC‐based search structure, search strings, limits, dates searched, and records identified for each database. Supplementary File S2 provides the completed PRISMA‐ScR checklist, which reports the location of each reporting item in the manuscript and supplementary materials.
