# Supplementary material for: Digital and Smart Technologies for Early Pressure Injury Detection and Prevention in Stroke and Mobility‐Impaired Patients: A Scoping Review
Source: Nurs Res Pract. 2026 Aug 12;2026:6992856. doi: 10.1155/nrp/6992856 (PMC13463426; doi:10.1155/nrp/6992856)
Supplement: Supplementary file 1 [file NRP-2026-6992856-s001.zip › Supplementary File S2 PRISMA ScR Checklist_2.docx]

**Supplementary File S2. PRISMA-ScR Checklist**

**Note.** This checklist is based on the PRISMA extension for Scoping Reviews (PRISMA-ScR) and is completed for the manuscript entitled "Digital and Smart Technologies for Early Pressure Injury Detection and Prevention in Stroke and Mobility-Impaired Patients: A Scoping Review." Page numbers should be updated after journal formatting if required by the submission system.

| **Item** | **PRISMA-ScR checklist item** | **Reported** | **Location in manuscript** | **Notes** |
| --- | --- | --- | --- | --- |
| 1 | Title: Identify the report as a scoping review. | Yes | Title page and manuscript title | The title explicitly identifies the work as a scoping review. |
| 2 | Structured summary: Provide a structured summary including background, objectives, eligibility criteria, sources of evidence, charting methods, results, and conclusions related to the review questions. | Yes | Abstract | The abstract reports background, methods, results, conclusion, and implications. |
| 3 | Rationale: Describe the rationale for the review in the context of what is already known and explain why a scoping review approach is appropriate. | Yes | Introduction; Methods - Design | The manuscript explains fragmented evidence across technologies, settings, and populations and states that evidence mapping was the objective. |
| 4 | Objectives: Provide an explicit statement of the questions and objectives being addressed with reference to their key elements. | Yes | Objectives | The PCC framework and four review questions are reported. |
| 5 | Protocol and registration: Indicate whether a review protocol exists and whether it is available or registered. | Yes | Methods - Protocol and registration | The manuscript states that no publicly accessible protocol or registration record was available and that methods were developed a priori. |
| 6 | Eligibility criteria: Specify characteristics of sources of evidence used as eligibility criteria and provide a rationale. | Yes | Methods - Eligibility criteria | Eligibility criteria include population, technology concept, prevention pathway relevance, study designs, publication period, language, and exclusion criteria. |
| 7 | Information sources: Describe all information sources in the search and the date of the most recent search. | Yes | Methods - Search strategy and information sources; Supplementary Table S1 | Databases and date searched are reported; search was completed on 30 July 2025. |
| 8 | Search: Present the full electronic search strategy for at least one database, including any limits used. | Yes | Methods - Search strategy and information sources; Supplementary Table S1 | Full PubMed/MEDLINE strategy and database-specific adaptations are provided in Supplementary Table S1. |
| 9 | Selection of sources of evidence: State the process for selecting sources of evidence included in the scoping review. | Yes | Methods - Study selection; Results - Study selection; Figure 1 | Two reviewers screened records using Rayyan; full-text assessment and resolution of disagreements are described. |
| 10 | Data charting process: Describe the methods of charting data from included sources of evidence and any processes used to ensure consistency. | Yes | Methods - Data charting | A modified JBI charting form was used and discussed by the review team before extraction. |
| 11 | Data items: List and define all variables for which data were sought and any assumptions or simplifications made. | Yes | Methods - Data charting; Table 1 | Charted items include author/year, country, aim, design, population/sample, setting, technology type, prevention-related function, outcomes, and implementation notes. |
| 12 | Critical appraisal of individual sources of evidence: If done, provide rationale and methods for critical appraisal. | Not applicable / Not undertaken | Methods - Critical appraisal; Limitations | Formal critical appraisal was not undertaken because the purpose was evidence mapping rather than effect estimation. |
| 13 | Synthesis of results: Describe methods of handling and summarizing the data that were charted. | Yes | Methods - Data synthesis | The manuscript describes descriptive synthesis and function-based thematic synthesis. |
| 14 | Selection of sources of evidence: Give numbers of sources screened, assessed for eligibility, and included, with reasons for exclusions at each stage. | Yes | Results - Study selection; Figure 1 | The PRISMA-ScR flow diagram reports identification, duplicate removal, screening, full-text assessment, exclusions, and inclusion. |
| 15 | Characteristics of sources of evidence: Present characteristics of each included source of evidence. | Yes | Results - Characteristics of included studies; Table 1 | Table 1 summarizes study characteristics and data charting results for all included studies. |
| 16 | Critical appraisal within sources of evidence: If done, present data on critical appraisal of included sources. | Not applicable | Methods - Critical appraisal; Limitations | No formal critical appraisal was conducted; this is acknowledged as a limitation and findings are interpreted descriptively. |
| 17 | Results of individual sources of evidence: For each included source, present relevant data that were charted. | Yes | Table 1 | Table 1 presents technology type, dominant prevention-related function, reported outcomes/key findings, and prevention-related implications. |
| 18 | Synthesis of results: Summarize and/or present the charting results as they relate to the review objectives and questions. | Yes | Results - Technology categories and prevention-related functions; Function-based thematic synthesis; Evidence gaps; Figure 4 | The synthesis groups technologies according to dominant prevention-related function and identifies evidence gaps. |
| 19 | Summary of evidence: Summarize the main results, including an overview of concepts, themes, and types of evidence, and link to the review questions and objectives. | Yes | Discussion | Discussion summarizes technology patterns, SEM evidence concentration, heterogeneity, population/setting gaps, and implementation issues. |
| 20 | Limitations: Discuss limitations of the scoping review process. | Yes | Limitations | Limitations include database restriction, language/time limits, lack of grey literature, no quality appraisal, heterogeneity, and limited stroke-specific evidence. |
| 21 | Conclusions: Provide a general interpretation of results with respect to objectives and potential implications. | Yes | Conclusion; Clinical and Research Implications | Conclusions are framed cautiously and emphasize mapping, potential roles, heterogeneity, and research needs. |
| 22 | Funding: Describe sources of funding and the role of funders for the scoping review. | Yes | Title page - Funding | Funding source and funder role are reported. The manuscript states that the funder had no role in the design, search process, data charting, synthesis, interpretation, or writing of the scoping review. |

**Abbreviations:** JBI = Joanna Briggs Institute; PRISMA-ScR = Preferred Reporting Items for Systematic Reviews and Meta-Analyses extension for Scoping Reviews.
