# Supplementary material for: Digital and Smart Technologies for Early Pressure Injury Detection and Prevention in Stroke and Mobility‐Impaired Patients: A Scoping Review
Source: Nurs Res Pract. 2026 Aug 12;2026:6992856. doi: 10.1155/nrp/6992856 (PMC13463426; doi:10.1155/nrp/6992856)
Supplement: Supplementary file 1 [file NRP-2026-6992856-s001.zip › Supplementary Table S1 Search Strategy.docx]

**Supplementary Table S1. Full Search Strategy and Database-Specific Adaptations**

**PCC-based search strategy.** The search strategy was developed using the Population-Concept-Context (PCC) framework. The Population component included adults with stroke or mobility-related vulnerability, including patients described as having mobility impairment, limited mobility, immobility, being bedridden, wheelchair use, or spinal cord injury. The Concept component included digital and smart technologies, including artificial intelligence, mobile health, wearable devices, sensor-based systems, monitoring systems, Internet of Things-based systems, smart beds, thermography, and sub-epidermal moisture assessment. The Context component included pressure injury prevention and early prevention-related assessment, including early detection, monitoring, risk assessment, repositioning, and pressure redistribution. Database-specific syntax was adapted according to each platform while preserving these PCC components.

**Note.** This supplementary table reports the final search strategy used to identify records for the scoping review. Database-specific syntax was adapted according to each platform while preserving the same core concepts: digital/smart technology, pressure injury/pressure ulcer, mobility-vulnerable populations, and prevention-related functions. Searches were limited to English-language records published from 1 January 2018 to 30 July 2025. The record counts correspond to the identification stage reported in Figure 1 of the manuscript.

**Core search concepts derived from the PCC framework:** (1) stroke or mobility-impaired populations; (2) digital or smart technologies; and (3) pressure injury prevention or early prevention-related assessment, including detection, monitoring, repositioning, pressure redistribution, and risk assessment.

## PubMed/MEDLINE

| **Platform** | PubMed |
| --- | --- |
| **Search string** | (("pressure ulcer"[MeSH Terms] OR "pressure injury"[Title/Abstract] OR "pressure injuries"[Title/Abstract] OR "pressure ulcer"[Title/Abstract] OR "pressure ulcers"[Title/Abstract] OR bedsore*[Title/Abstract] OR "bed sore*"[Title/Abstract] OR "decubitus ulcer*"[Title/Abstract]) AND ("digital technology"[Title/Abstract] OR "smart technology"[Title/Abstract] OR "artificial intelligence"[MeSH Terms] OR "artificial intelligence"[Title/Abstract] OR "machine learning"[Title/Abstract] OR "mobile health"[Title/Abstract] OR mHealth[Title/Abstract] OR smartphone*[Title/Abstract] OR "wearable electronic devices"[MeSH Terms] OR "wearable device*"[Title/Abstract] OR sensor*[Title/Abstract] OR "monitoring system*"[Title/Abstract] OR "Internet of Things"[Title/Abstract] OR IoT[Title/Abstract] OR "smart bed*"[Title/Abstract] OR thermograph*[Title/Abstract] OR "subepidermal moisture"[Title/Abstract] OR "sub-epidermal moisture"[Title/Abstract] OR "SEM scanner"[Title/Abstract]) AND ("stroke"[MeSH Terms] OR stroke[Title/Abstract] OR poststroke[Title/Abstract] OR "post-stroke"[Title/Abstract] OR "mobility impairment"[Title/Abstract] OR "limited mobility"[Title/Abstract] OR immobile[Title/Abstract] OR immobilized[Title/Abstract] OR immobilised[Title/Abstract] OR bedridden[Title/Abstract] OR "spinal cord injury"[MeSH Terms] OR "spinal cord injur*"[Title/Abstract]) AND (prevention[Title/Abstract] OR prevent*[Title/Abstract] OR detection[Title/Abstract] OR monitoring[Title/Abstract] OR "risk assessment"[MeSH Terms] OR "risk assessment"[Title/Abstract] OR reposition*[Title/Abstract] OR "pressure redistribution"[Title/Abstract])) |
| **Limits/filters** | Publication date: 1 January 2018 to 30 July 2025; Language: English. |
| **Date searched** | 30 July 2025 |
| **Records identified** | n = 283 |
| **Adaptation note** | MeSH terms were combined with Title/Abstract keywords. The adult/mobility-impaired population criterion was applied during screening when not fully captured by database indexing. |

## ScienceDirect

| **Platform** | ScienceDirect |
| --- | --- |
| **Search string** | ("pressure injury" OR "pressure injuries" OR "pressure ulcer" OR "pressure ulcers" OR bedsore OR "bed sore" OR "decubitus ulcer") AND ("digital technology" OR "smart technology" OR "artificial intelligence" OR "machine learning" OR "mobile health" OR mHealth OR smartphone OR wearable OR sensor OR "monitoring system" OR "Internet of Things" OR IoT OR "smart bed" OR thermography OR "sub-epidermal moisture" OR "subepidermal moisture" OR "SEM scanner") AND (stroke OR poststroke OR "post-stroke" OR immobilized OR immobilised OR immobile OR "limited mobility" OR "mobility impairment" OR bedridden OR "spinal cord injury") AND (prevention OR detection OR monitoring OR "risk assessment" OR repositioning OR "pressure redistribution") |
| **Limits/filters** | Years: 2018 to 2025; Language: English. |
| **Date searched** | 30 July 2025 |
| **Records identified** | n = 703 |
| **Adaptation note** | Search terms were applied using ScienceDirect keyword search fields. Results were screened for empirical studies and relevance to pressure injury prevention-related technology. |

## CINAHL Complete via EBSCOhost

| **Platform** | EBSCOhost |
| --- | --- |
| **Search string** | (MH "Pressure Ulcer+" OR TI ("pressure injury" OR "pressure injuries" OR "pressure ulcer" OR "pressure ulcers" OR bedsore* OR "decubitus ulcer*") OR AB ("pressure injury" OR "pressure injuries" OR "pressure ulcer" OR "pressure ulcers" OR bedsore* OR "decubitus ulcer*")) AND (MH "Artificial Intelligence" OR MH "Mobile Applications" OR MH "Wearable Electronic Devices" OR TI ("digital technolog*" OR "smart technolog*" OR "artificial intelligence" OR "machine learning" OR "mobile health" OR mHealth OR smartphone* OR wearable* OR sensor* OR "monitoring system*" OR "Internet of Things" OR IoT OR "smart bed*" OR thermograph* OR "sub-epidermal moisture" OR "subepidermal moisture" OR "SEM scanner") OR AB ("digital technolog*" OR "smart technolog*" OR "artificial intelligence" OR "machine learning" OR "mobile health" OR mHealth OR smartphone* OR wearable* OR sensor* OR "monitoring system*" OR "Internet of Things" OR IoT OR "smart bed*" OR thermograph* OR "sub-epidermal moisture" OR "subepidermal moisture" OR "SEM scanner")) AND (MH "Stroke+" OR TI (stroke OR poststroke OR "post-stroke" OR immobilized OR immobilised OR immobile OR "limited mobility" OR "mobility impairment" OR bedridden OR "spinal cord injur*") OR AB (stroke OR poststroke OR "post-stroke" OR immobilized OR immobilised OR immobile OR "limited mobility" OR "mobility impairment" OR bedridden OR "spinal cord injur*")) AND (TI (prevention OR prevent* OR detection OR monitoring OR "risk assessment" OR reposition* OR "pressure redistribution") OR AB (prevention OR prevent* OR detection OR monitoring OR "risk assessment" OR reposition* OR "pressure redistribution")) |
| **Limits/filters** | Publication date: 2018 to 2025; Language: English. |
| **Date searched** | 30 July 2025 |
| **Records identified** | n = 6 |
| **Adaptation note** | CINAHL Headings and title/abstract terms were combined to capture nursing and allied health literature. |

## IEEE Xplore

| **Platform** | IEEE Xplore |
| --- | --- |
| **Search string** | (("pressure injury" OR "pressure ulcer" OR bedsore OR "decubitus ulcer") AND ("digital technology" OR "smart technology" OR "artificial intelligence" OR "machine learning" OR "mobile health" OR smartphone OR wearable OR sensor OR "monitoring system" OR "Internet of Things" OR IoT OR "smart bed" OR thermography OR "sub-epidermal moisture" OR "SEM scanner") AND (stroke OR immobilized OR immobilised OR immobile OR "limited mobility" OR "mobility impairment" OR bedridden OR "spinal cord injury") AND (prevention OR detection OR monitoring OR "risk assessment" OR repositioning OR "pressure redistribution")) |
| **Limits/filters** | Publication years: 2018 to 2025; English-language records where available. |
| **Date searched** | 30 July 2025 |
| **Records identified** | n = 15 |
| **Adaptation note** | Search terms were applied to metadata/full-text search fields to capture engineering, sensor, mobile, artificial intelligence, and Internet of Things technologies relevant to pressure injury prevention. |

## Wiley Online Library

| **Platform** | Wiley Online Library |
| --- | --- |
| **Search string** | ("pressure injury" OR "pressure ulcer" OR bedsore OR "decubitus ulcer") AND ("digital technology" OR "smart technology" OR "artificial intelligence" OR "machine learning" OR "mobile health" OR smartphone OR wearable OR sensor OR "monitoring system" OR "Internet of Things" OR IoT OR "smart bed" OR thermography OR "sub-epidermal moisture" OR "SEM scanner") AND (stroke OR immobilized OR immobilised OR immobile OR "limited mobility" OR "mobility impairment" OR bedridden OR "spinal cord injury") AND (prevention OR detection OR monitoring OR "risk assessment" OR repositioning OR "pressure redistribution") |
| **Limits/filters** | Publication years: 2018 to 2025; Language: English where available. |
| **Date searched** | 30 July 2025 |
| **Records identified** | n = 56 |
| **Adaptation note** | Boolean concepts were adapted to the Wiley Online Library search interface. Screening was used to exclude non-empirical or non-relevant records. |

**Record summary:** PubMed/MEDLINE (n = 283), ScienceDirect (n = 703), CINAHL Complete via EBSCOhost (n = 6), IEEE Xplore (n = 15), and Wiley Online Library (n = 56), for a total of 1,063 records identified before duplicate removal.
